# Massachusetts Veterinary Association

**Published:** 1894-09

**Authors:** John M. Parker

**Affiliations:** Secretary


### MASSACHUSETTS VETERINARY ASSOCIATION.

*January.*

The regular monthly meeting of the Massachusetts Veterinary Association was held at 19 Boylston Place, on January 24, 1894, at seven-thirty P. M.

The President, Dr. Burr, occupied the chair. The members present were: Drs. Becket, Blackwood, Burr, Emerson, Towle, Howard Marshall, Parker, Simpson, Winchester and Winslow. Honorary member, Dr. Stickney.

The minutes of the last meeting were then read and accepted, and there being no business before the Association, and the regular essayist not having arrived, Dr. Parker read a paper on "The Prevention of Tuberculosis." (Vide March No., page 182 this journal.

In the discussion which followed Dr. Winchester suggested that the Association take steps to have tuberculosis placed on the list of contagious diseases. It is generally accepted that there is no other disease which causes so many deaths as tuberculosis in its various forms. As showing the value of sanitary reform, he referred to the towns in the Merrimac Valley. These towns use the river water for public services. Through the exertions of one of the members of the State Board of Health, a filtering gallery for river water was put in at the City of Lawrence; since then the death rate has been much reduced. The death rate from typhoid is now under 4 per cent., while the death rate from tuberculosis is nearly 8 per cent. Continuing, he said this Association ought to be the first to put itself on record as recognizing tuberculosis as a contagious disease.

*Dr. Emerson* thinks the idea of conferring with the State Board of Health is a good one.

*Dr. Becket* said he had been somewhat surprised lately to see in insurance reports an article congratulating the companies and the public on the fact that, according to the insurance reports, people are getting longer lived, and that deaths from tuberculosis are gradually decreasing.

*Dr. Parker* stated that the reason given for the seeming falling off in deaths from tuberculosis was because the establishment of post-graduate schools, and the consequent better education of country practitioners, had resulted in greater strictness in examination; fewer poor risks had been taken by insurance companies, with the result that in insurance commission reports the death rate from tuberculosis had decreased.

After a further discussion Dr. Winchester moved that a committee be appointed from members of the Association to approach the authorities on the subject.

*Dr. Burr* wished to point out that in the city of Boston the by-laws required that each animal shall have 1,000 c.f. Many of the other points suggested by the essayist were at present in force in the city. He thought the cattle commissioners were doing all in their power with the force at their disposal. The amount of work they had to do was very great.

*Dr. Howard* thinks if anything is to be done in the matter it should be well discussed, statistics should be looked up, and the Association should be sure of its ground. He then moved that the discussion should be continued at the next meeting. Seconded by Dr. Winslow.

*Dr. Parker* moved, as an amendment, that a committee be appointed, consisting of members of the Association who have had experience with tuberculosis, to report at next meeting on the subject of tuberculosis. Seconded by Dr. Blackwood and carried unanimously.

*Dr. Winslow* then moved that the committee consist of five persons, with the President and Secretary ex-officio. Carried.

*Dr. Howard* moved that the committee be appointed from the floor. Carried. The following committee were then appointed, with instructions to report at next meeting: Dr. James B. Paige, Amherst; Dr. Winchester, Lawrence; Drs. Peters, Howard and Osgood, Boston with the President and Secretary ex-officio.

*Dr. Simpson* then read an interesting paper on "Digitalis"

In the discussion which followed Dr. Howard said he usually tried to limit the amount given to 1 drachm of the fluid extract in the twenty-four hours. The great danger in its use is because of the digestive troubles that supervene. It should be used with caution. When judiciously used, however, it is a valuable remedy.

*Dr. Becket* has had good results where the horse has recovered from the acute stage of pneumonia, leaving him with feeble pulse. It should, however, be used with great caution.

*Dr. Winchester* has had good results from veratrum viride In cases where the pulse was 80 or 90, and small and weak, he has used veratrum, and in a few hours the pulse has come away down and is strong and full ; afterwards puts on to alcohol. The after results of "Digitalis" are sometimes serious, but has never seen any bad results from veratrum.

After a good deal of discussion, and a vote of thanks to the essayist, the President called for reports of cases.

*Dr. Parker* reported an unusual case of azoturea. The horse belonged to the Haverhill St R. R. Co., and had not had a day off for three years. He was taken sick at the end of a day's work, when he was on his last trip—about five miles from his stable, he was noticed to stumble once or twice ; finally he came down, unable to use his sore legs. The day was very stormy, sleet was falling and a nasty wind was blowing. The horse unfortunately had to lie on the wet snow in the storm for about two hours, covered only by a couple of old sacks; finally he was put on a stone drag, and hauled to a barn one-half mile away.

He was suffering a good deal; the muscles of the shoulders were rigid; he was unable to use his fore legs ; and his urine was dark and coffee-colored. The next morning, with the help of slings, he was placed on his feet, put on a sled, and taken home—where he died the following night.

*Dr. Howard* reported a somewhat similar case, where the horse had been working every day, and had not been loafing. When seen he was unable to stand, and the urine had the characteristic dark color. He recollected three cases with paralysis forward, muscles of the shoulder hard, and dark-colored urine.

*Dr. Blackwood* has had several cases where the fore extremity had been affected. In these cases it is common to have atrophy of the muscles.

*Dr. Marshall* reported a case where the horse recovered from the first attack, and for some time afterward he had periodical weekly attacks.

Some of the other members have had similar cases to those reported.

The discussion then turned on kicking mares, and the effect produced by spaying and excism of the clitoris. Dr. Becket reported three cases in which he had excised the clitoris by making an incision round the base and sloughing off by common elastic band. He had good results in two of the cases mentioned.

The meeting then adjourned till next month.

JOHN M. PARKER, Secretary.

*February.*

The regular monthly meeting of the Massachusetts Veterinary Association was held at 19 Boylston Place, on Wednesday, February 28, 1894, at seven-thirty P M. The members present were Drs. Becket, Bryden, Burr, Emerson, Howard, Labaw, Marshall, Osgood, Parker, Peterson. Soule, Winchester and Winslow. Honorary member, Dr. Stickney.

The minutes of the last meeting having been read and accepted, the report of the Committee on Tuberculosis was submitted.

The recommendation of the committee embraced the following points:

1st.—That the March meeting be devoted to the consideration of the subject of tuberculosis.

2d.—If the previous recommendation be adopted by the Association, that the members of the Association invite any person interested in the subject of tuberculosis to be present.

3d —That the Association employ a stenographer for the evening of the March meeting.

4th.—That the Association pass a resolution putting themselves on record as recommending that, as Bovine Tuberculosis has been placed under the "Contagious Diseases of Animals" Act, we believe that certain forms of the disease in man should be so recognized.

The *first recommendation*, that the March meeting be devoted to the discussion on the subject of "Tuberculosis," received a good deal of discussion. It was finally decided that the time before the March meeting was too short for the preparation of papers on such a subject. and it was thought best to delay the discussion till the month of May.

The *second recommendation*, that members invite any person interested in tuberculosis to be present, was unanimously adopted.

The consideration of the third recommendation was postponed till later.

The *fourth recommendation* created a lively discussion; Drs. Bryden, Howard, Osgood, Winchester and others taking part. Dr. Bryden objected to the passage of such a resolution on the ground that tuberculosis is neither so widespread nor so serious as has been represented Finally Dr. Howard offered the following resolution, which was seconded by Dr. Winchester:

*Resolved*, that as Bovine Tuberculosis is recognized by the "Contagious Diseases of Animals" Act as a contagious disease, in our opinion that, being due to the same germ, certain forms of the disease in *man* should *also* be recognized by law as contagious.

After some further discussion the resolution was adopted with one dissenting vote.

Drs. Osgood, Burr and Parker were then appointed a committee to interview some of the well known physicians and pathologists with the object of getting them to read short papers on tuberculosis at the May meeting.

There being no further business the meeting adjourned till further notice.

JOHN M. PARKER, Secretary.

